# Supplementary material for: RNA structure prediction using positive and negative evolutionary information
Source: PLoS Comput Biol. 2020 Oct 30;16(10):e1008387. doi: 10.1371/journal.pcbi.1008387 (PMC7657543; doi:10.1371/journal.pcbi.1008387)
Supplement: S1 File — Includes alignments, and source code. (GZ) [file pcbi.1008387.s008.tar.gz › supplemental_material/results/Figures/chrB.R2R.sto.pdf]

chrB

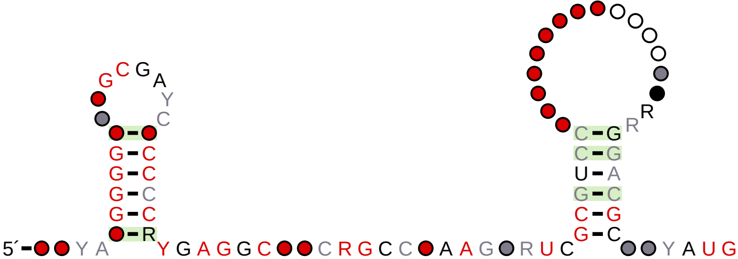
G C G A
Y
C
G-C
G-C
G-C
G-C
R
5´
Y A
Y G A G G C
C R G C C
A A G
R U C
R
R
C-G
C-G
U-A
G-C
C-G
G-C
Y A U G
